# Obturator Dislocation During Pavlik Harness Treatment for Developmental Dysplasia of the Hip: A Rare Complication and Its Clinical Course

**DOI:** 10.7759/cureus.106492

**Published:** 2026-04-05

**Authors:** Milena Bogojevska Doksevska, Danica Popovska, Smiljana Bundovska Kocev, Kristina Jordanoski, Biljana Lazarova

**Affiliations:** 1 Faculty of Medicine, Ss. Cyril and Methodius University, Skopje, MKD; 2 Orthopedic Surgery, University Clinic for Orthopedic Diseases, Skopje, MKD; 3 Radiology, University Institute of Radiology, Skopje, MKD; 4 Orthopedic Surgery, General Hospital, Strumica, MKD

**Keywords:** avascular necrosis of the hip, developmental dysplasia of the hip, obturator (inferior) dislocation, pavlik harness, persistent dysplasia

## Abstract

Developmental dysplasia of the hip represents a spectrum of disorders ranging from mild acetabular dysplasia to complete hip dislocation. Early diagnosis and treatment are essential to prevent long-term complications. The Pavlik harness remains the first-line treatment for infants with reducible hip dysplasia or dislocation diagnosed within the first months of life and has demonstrated high success rates. However, complications related to its use may occasionally occur. One of the rare but significant complications is obturator (inferior) dislocation of the hip during treatment.

We present the case of a two-month-old female infant diagnosed with Graf type IV developmental dysplasia of the right hip. Following reduction and treatment with a Pavlik harness, follow-up imaging revealed an obturator dislocation of the hip. The harness was discontinued, and closed reduction with subsequent casting was performed. Despite an initially successful reduction, the patient later developed persistent dysplasia, subluxation, and femoral head fragmentation. Long-term follow-up demonstrated improvement in hip stability, but residual acetabular dysplasia and deformity of the ossific nucleus were present, consistent with coxa plana. The patient is currently scheduled for a corrective pelvic osteotomy.

This case highlights the importance of careful monitoring during Pavlik harness treatment and early recognition of atypical displacement patterns. Prompt diagnosis and appropriate management are essential to prevent further complications and optimize long-term hip development.

## Introduction

Developmental dysplasia of the hip (DDH) encompasses a range of abnormalities involving the acetabulum and proximal femur, varying from mild acetabular dysplasia to complete hip dislocation. It is one of the most common musculoskeletal conditions in infants. The incidence of hip dislocation is estimated at approximately one to two per 1,000 live births, while hip instability may occur in up to 10 per 1,000 live births [1-3]. Early detection is crucial because untreated DDH can lead to long-term complications such as gait abnormalities, chronic pain, limb length discrepancy, and early degenerative osteoarthritis. Consequently, screening programs have been widely implemented to allow early diagnosis and intervention.

DDH encompasses a range of abnormalities involving the acetabulum and proximal femur, varying from mild acetabular dysplasia to complete hip dislocation. It is one of the most common musculoskeletal conditions in infants. The incidence of hip dislocation is estimated at approximately one to two per 1,000 live births, while hip instability may occur in up to 10 per 1,000 live births [1-3]. Early detection is crucial because untreated DDH can lead to long-term complications such as gait abnormalities, chronic pain, limb length discrepancy, and early degenerative osteoarthritis. Consequently, screening programs have been widely implemented to allow early diagnosis and intervention.

Clinical screening primarily relies on physical examination using the Ortolani and Barlow maneuvers, which are designed to identify hip instability in newborns and young infants [4,5]. Imaging studies complement clinical evaluation and play a crucial role in confirming the diagnosis. Ultrasonography has become the preferred diagnostic method in infants younger than six months of age because it allows dynamic evaluation of the hip joint before ossification of the femoral head occurs. The Graf classification system is widely used to categorize sonographic findings and guide treatment decisions [6]. When DDH is detected early, conservative treatment can be initiated during the period of greatest hip joint plasticity. The Pavlik harness is widely considered the first-line treatment for infants with reducible hip dysplasia or dislocation diagnosed within the first months of life. The harness maintains the hips in flexion and controlled abduction, allowing the femoral head to remain concentrically reduced within the acetabulum while preserving active movement. This positioning promotes normal acetabular development and stabilization of the hip joint [7,8]. When properly applied, the Pavlik harness has success rates exceeding 90% and significantly reduces the need for surgical intervention [9]. Nevertheless, complications may occasionally arise during treatment, including femoral nerve palsy, avascular necrosis, or atypical patterns of hip displacement [10-13]. Obturator dislocation of the hip during Pavlik harness treatment is an uncommon complication but may lead to a cascade of further problems if not promptly recognized.

To our knowledge, only five case reports consisting of seven patients with iatrogenic obturator dislocation have been published, with no series available [10-12]. There is no theory on the pathophysiology of this complication: is it because of hyperlaxity or excessive dysplasia of the hip, or is it caused iatrogenically? Due to the low occurrence of this complication, there is not enough data to be analyzed. The present report describes such a case and outlines its subsequent clinical course.

## Case presentation

**Figure 1:**
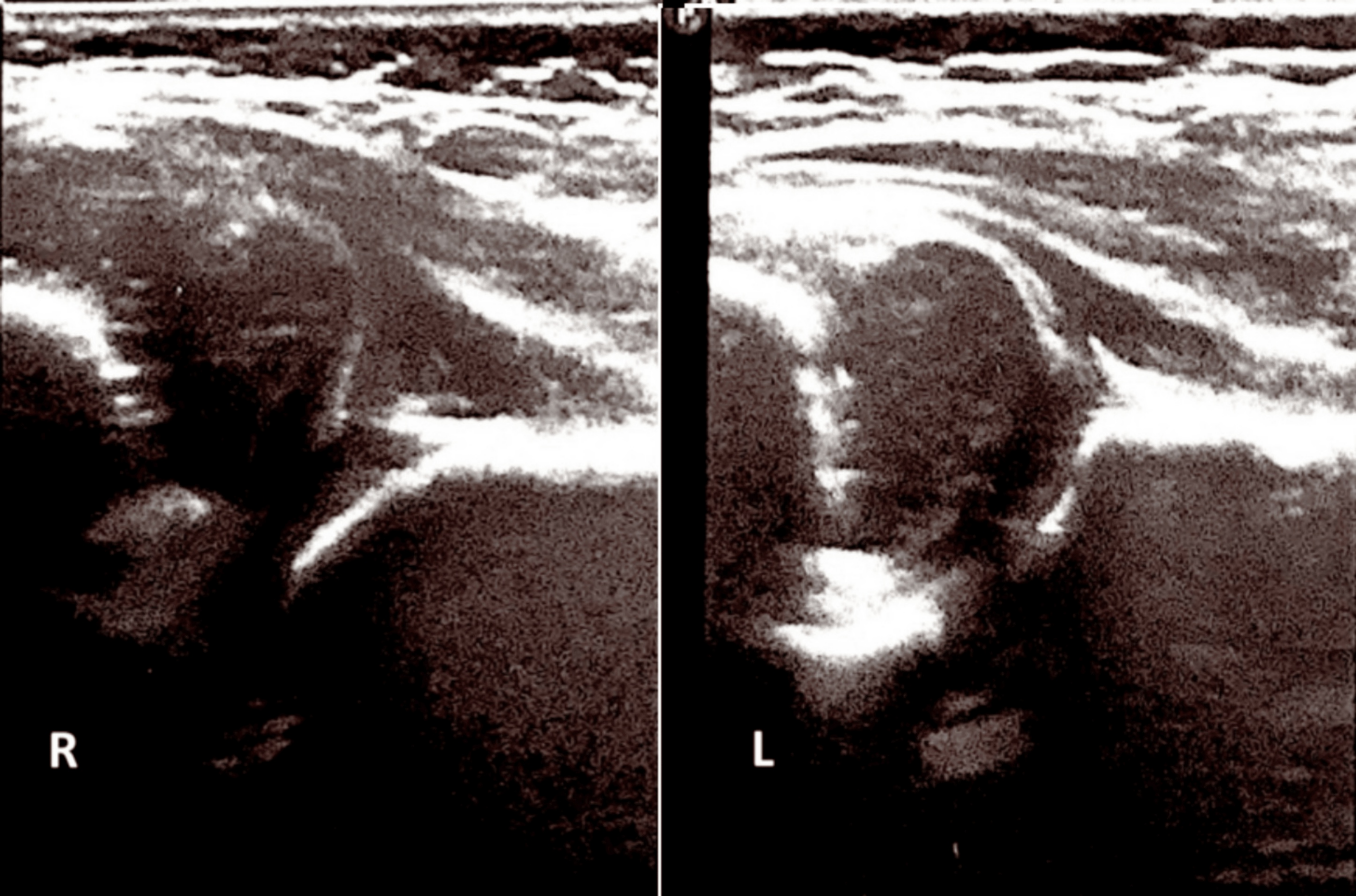
Ultrasound image demonstrating right hip dislocation in the developmental dysplasia of the hip. R: right. L: left

**Figure 2:**
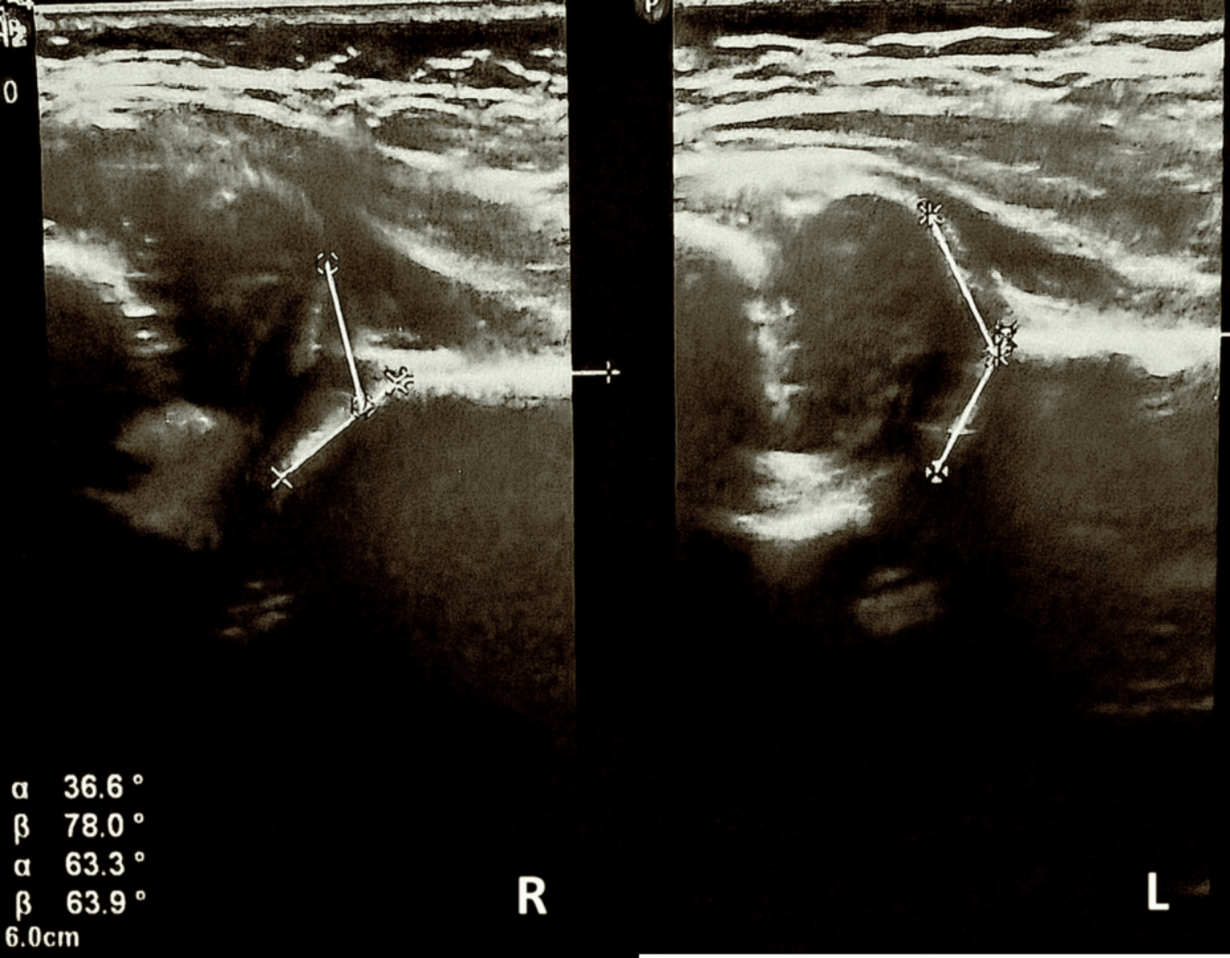
Graf ultrasound measurements demonstrating type IV hip dysplasia with a decreased alpha angle and an increased beta angle on the right side, on the left side the angles are normal. R: right. L: left

A two-month-old female infant presented to our outpatient clinic as part of a routine screening program for developmental dysplasia of the hip. Physical examination revealed reduced abduction of the right hip with positive Ortolani and Barlow signs. The infant was the first child, born at term after an uncomplicated pregnancy. There was no family history of hip dysplasia. The remainder of the musculoskeletal examination was unremarkable. Ultrasonographic evaluation of the hips using the Graf method demonstrated type IV hip dysplasia on the right side, with an alpha angle of 36.6° and a beta angle of 78° (Figures 1, 2). The left hip showed normal clinical and ultrasonographic findings. The proximal femoral epiphyseal nucleus was not yet visible on either side, which was appropriate for the patient’s age.

**Figure 3:**
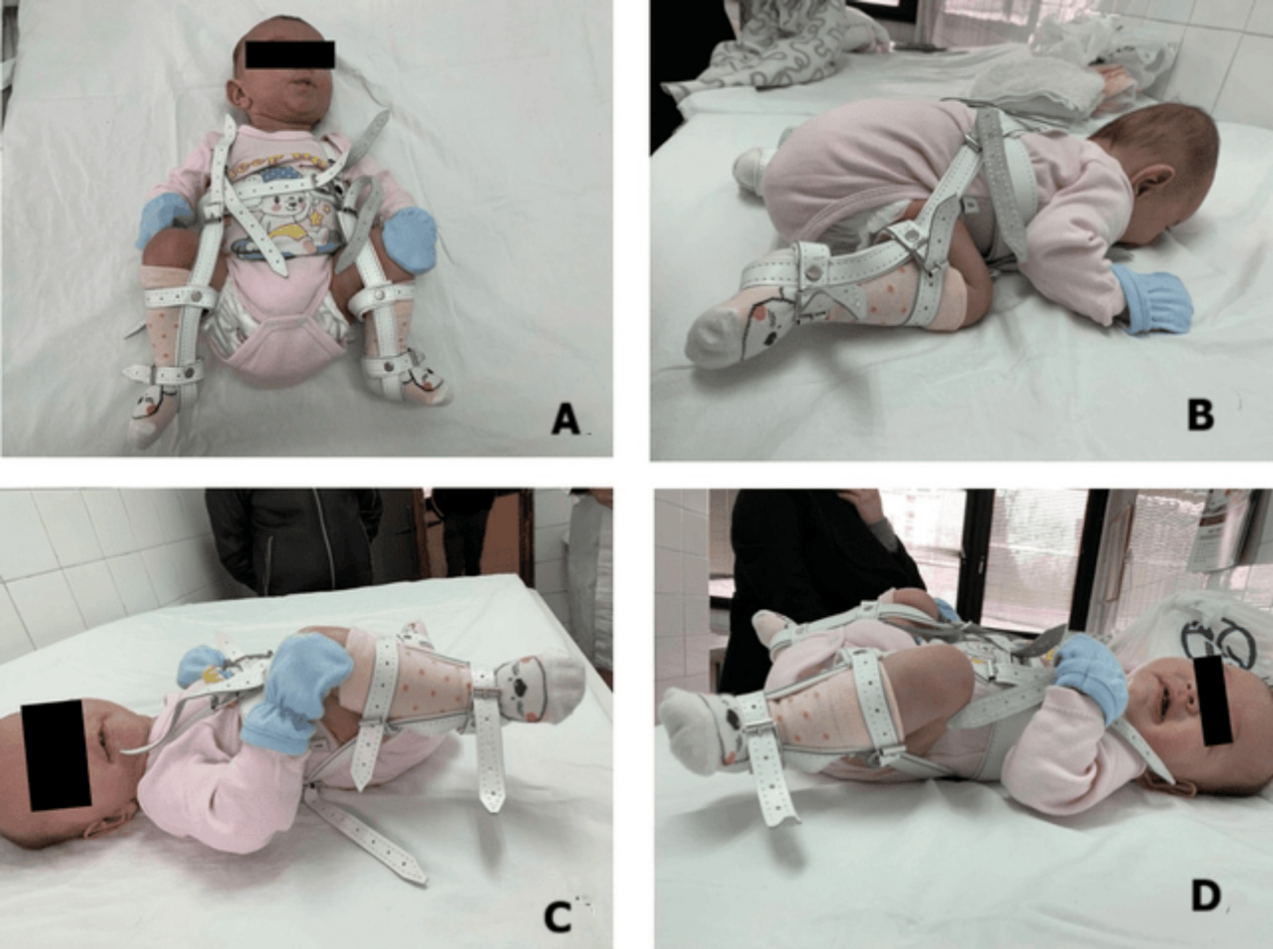
Clinical photographs showing the patient treated with a Pavlik harness. Note the increased hip flexion used to maintain the reduction. (A) Anterior supine view. (B) Prone view. (C) Left lateral supine view. (D) Right lateral supine view

Following reduction of the hip using the Ortolani maneuver, a Pavlik harness was applied with limited abduction and increased flexion to maintain the reduction (Figure 3). The parents were instructed not to remove the harness during the first week of treatment.

**Figure 4:**
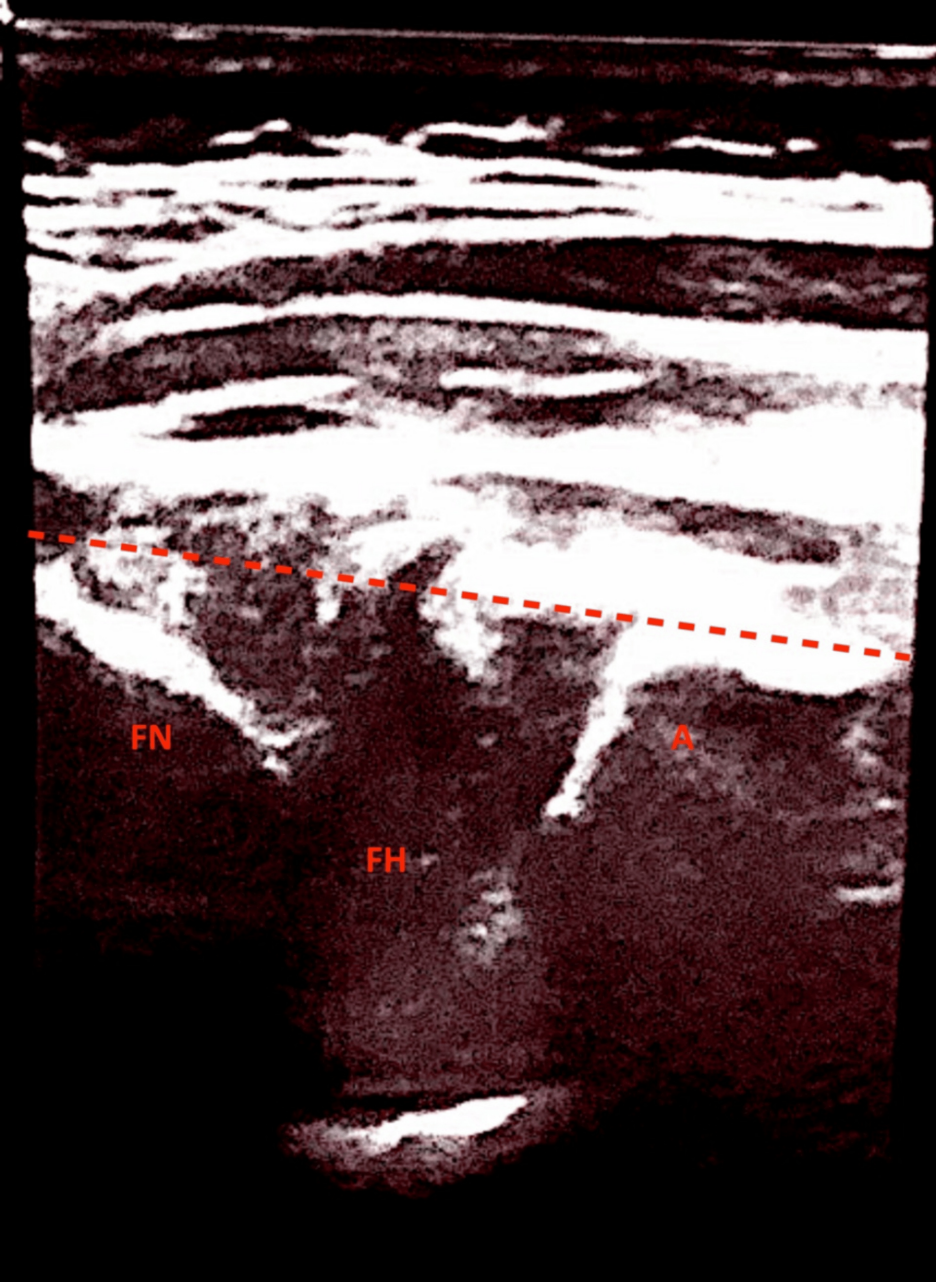
Anterior hip ultrasound using the van Douveren method, demonstrating disruption of the modified Shenton line, indicating hip dislocation A: acetabulum; FH: femoral head; FN: femoral neck

**Figure 5:**
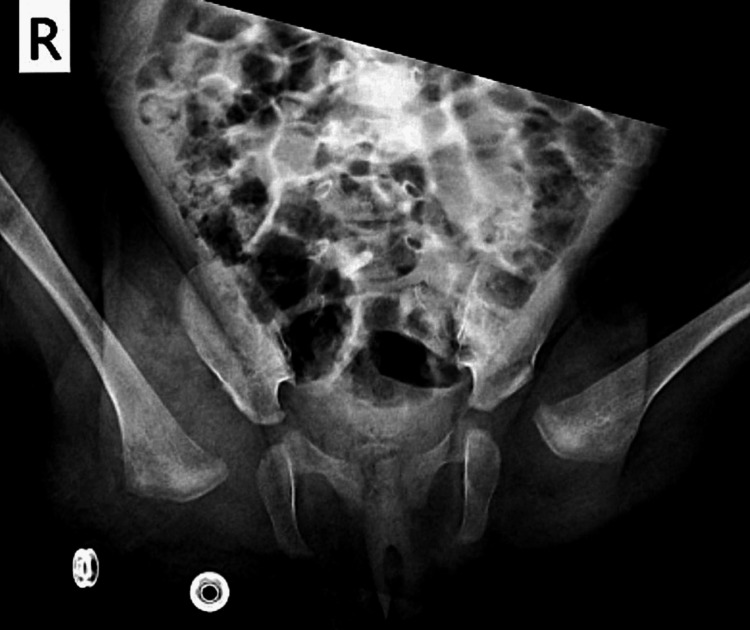
Anteroposterior pelvic radiograph demonstrating obturator (inferior) dislocation of the right hip

At the first follow-up appointment, one week after harness application, anterior hip ultrasonography was performed using the van Douveren method [14-16]. The parents reported that the infant had been restless and had developed redness and irritation in the inguinal folds. Ultrasound examination demonstrated disruption of the modified Shenton line, as described by Smith et al., suggesting hip dislocation (Figure 4) [15]. A plain pelvic radiograph was obtained and revealed an obturator (inferior) dislocation of the right hip (Figure 5).

**Figure 6:**
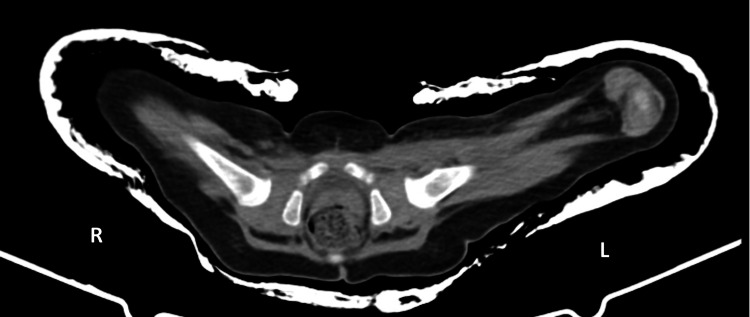
Postreduction axial CT scan confirming concentric reduction of the femoral head within the acetabulum. R: right. L: left

The Pavlik harness was immediately discontinued, and the patient was scheduled for closed reduction, which was performed using the standard technique. Postoperative limited CT imaging confirmed satisfactory reduction of the femoral head within the acetabulum (Figure 6). A hip spica cast was applied and maintained for three months, with one cast change during this period. The postoperative course was uneventful.

**Figure 7:**
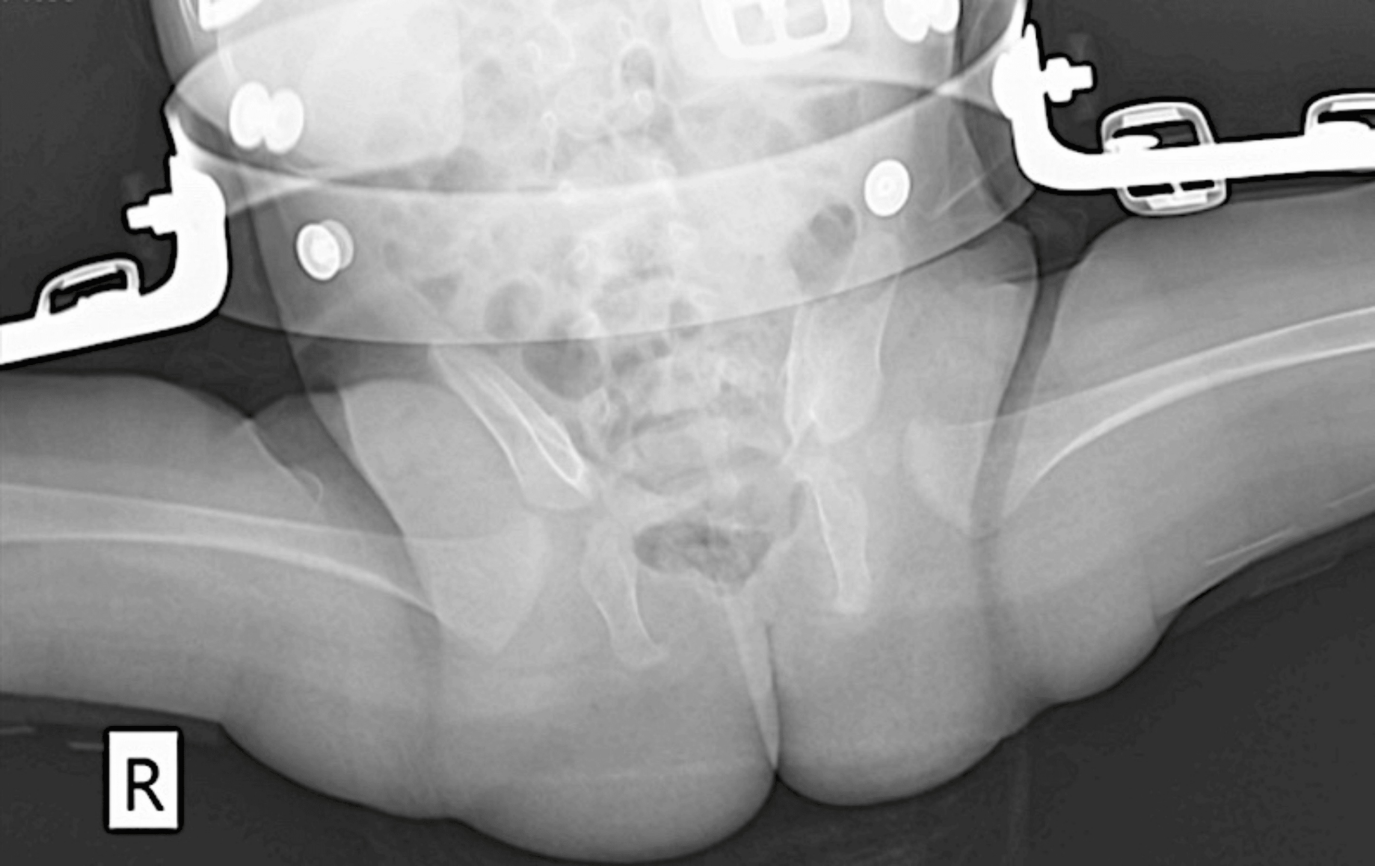
Pelvic radiograph demonstrating hip stabilization using a Hilgenreiner abduction brace

**Figure 8:**
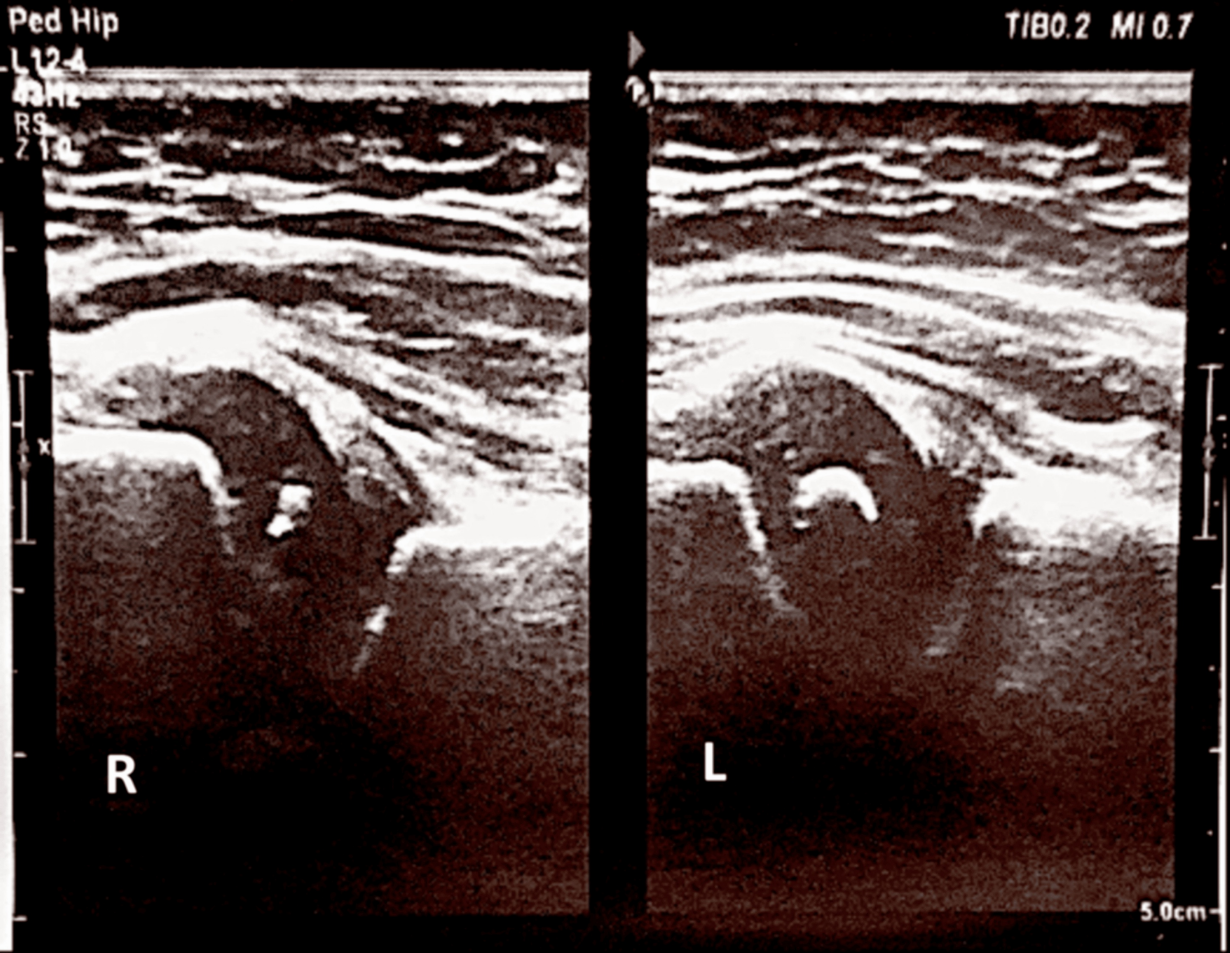
Follow-up hip ultrasound showing satisfactory reduction and appearance of the femoral head ossification nucleus. R: right. L: left

After removal of the cast, a Hilgenreiner abduction brace was applied (Figure 7). Subsequent ultrasonographic evaluation demonstrated stable hip reduction and the appearance of the femoral head ossification nucleus (Figure 8), after which the brace was discontinued.

**Figure 9:**
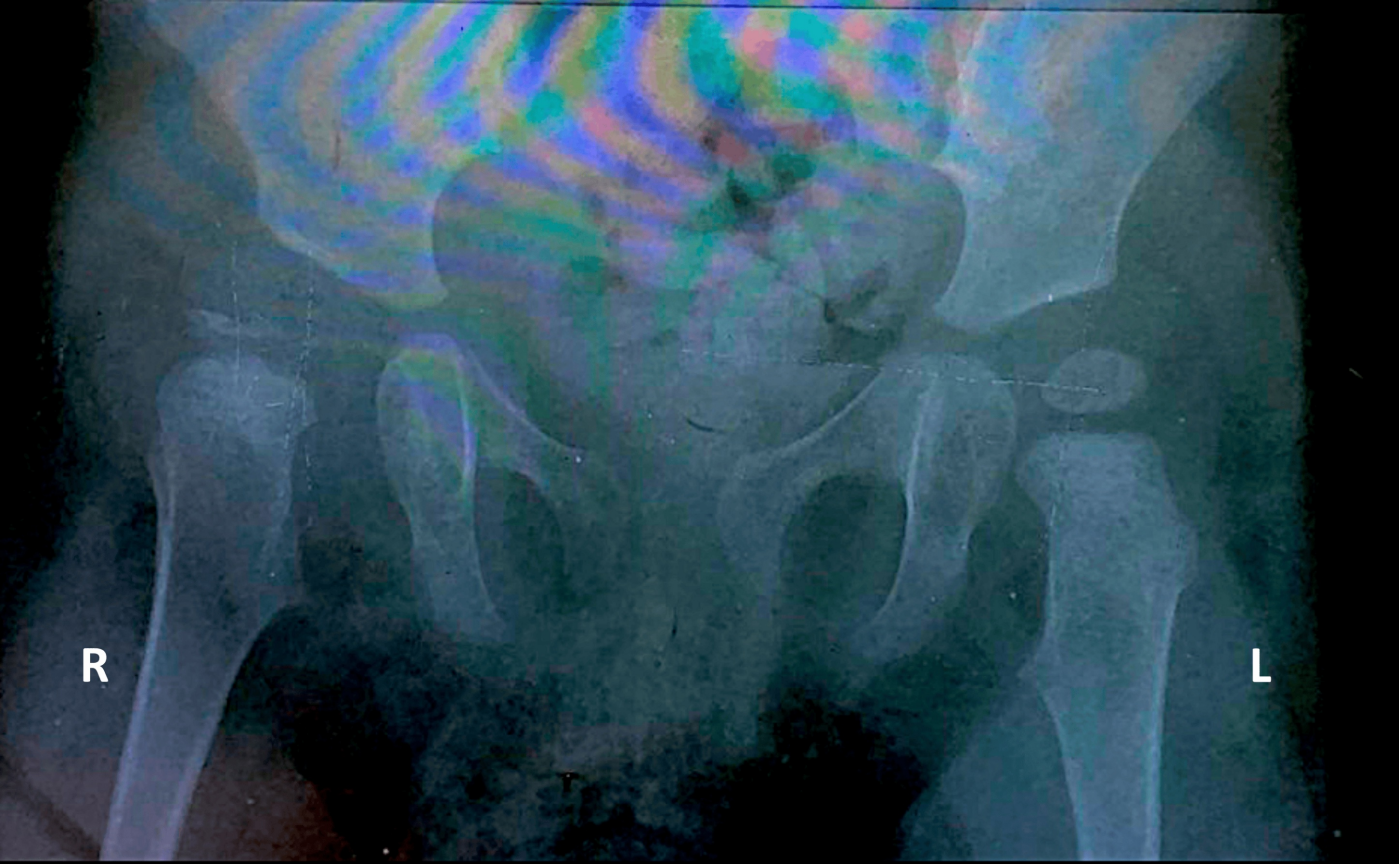
Follow-up radiograph demonstrating persistent acetabular dysplasia, hip subluxation, and fragmentation of the femoral head ossific nucleus (Tönnis and Kuhlmann type III). R: right. L: left

**Figure 10:**
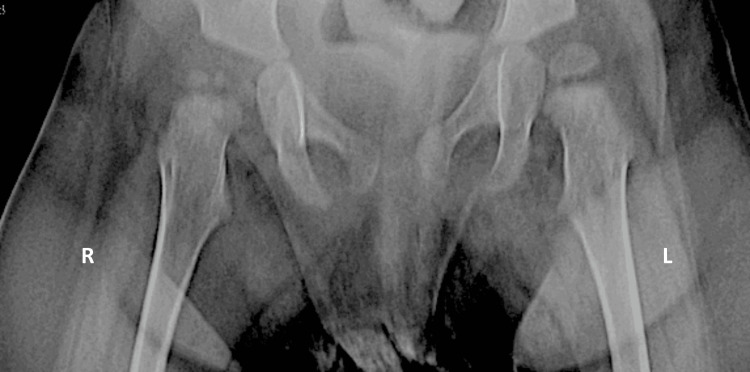
Radiograph obtained prior to discontinuation of the abduction brace showing improved hip positioning but persistent femoral head fragmentation. R: right. L: left

One month later, follow-up radiography revealed persistent acetabular dysplasia, hip subluxation, and fragmentation of the femoral head ossific nucleus, indicating avascular necrosis, consistent with Tönnis and Kuhlmann type III changes (Figure 9). At this stage, treatment with the abduction brace was reintroduced and continued for an additional three months, after which it was discontinued (Figure 10). During the entire treatment period, the patient achieved developmental milestones appropriate for age.

**Figure 11:**
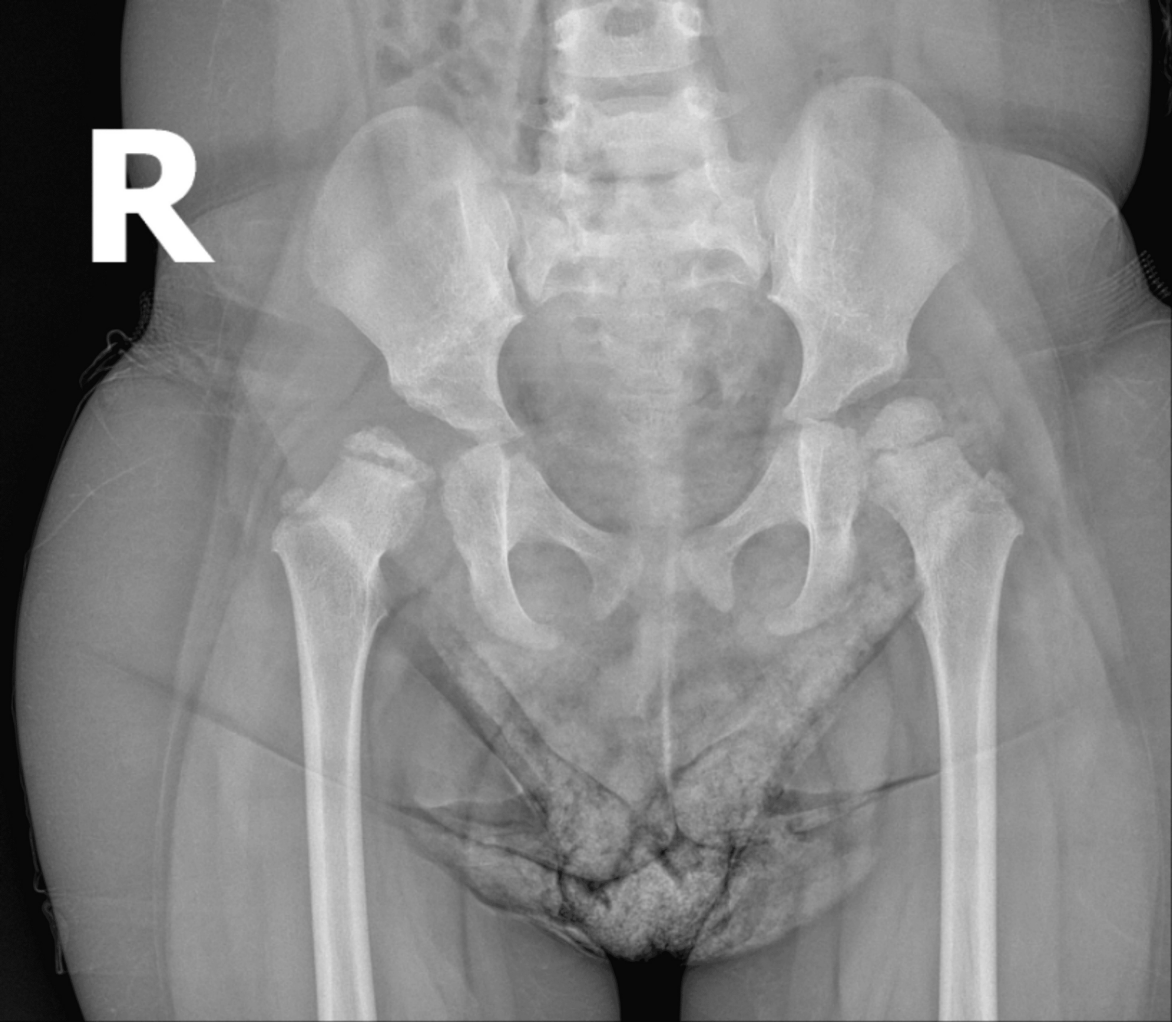
Two-year follow-up radiograph demonstrating hip subluxation, disrupted Shenton-Menard line, and persistent acetabular dysplasia

Clinical and radiographic follow-up was performed every six months. Two years after the initial treatment, the patient presented with a mild, painless limp. The Galeazzi sign was positive. Radiographs demonstrated disruption of the Shenton-Menard line, hip subluxation, and persistent acetabular dysplasia (Figure 11). At that time, no surgical intervention was planned.

**Figure 12:**
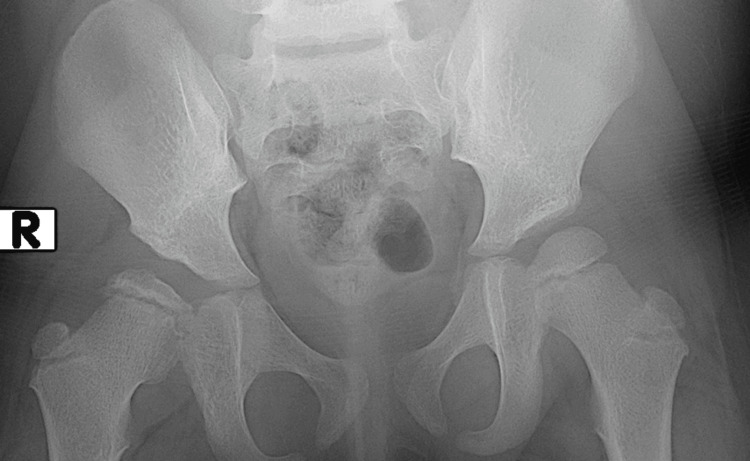
Most recent follow-up radiograph demonstrating restored Shenton-Menard line, deformity of the femoral head consistent with coxa plana, and increased acetabular index

At the most recent follow-up examination, the limp had resolved, and the hip was painless. Radiographic assessment demonstrated restoration of the Shenton-Menard line and an International Hip Dysplasia Institute (IHDI) classification type I. The ossific nucleus had resumed ossification but appeared deformed, consistent with coxa plana (Figure 12). The acetabular index remained elevated.

The patient is currently scheduled for Salter innominate osteotomy to correct the residual acetabular dysplasia.

## Discussion

The Pavlik harness is widely recognized as the standard first-line treatment for DDH diagnosed in early infancy. When applied correctly and monitored appropriately, it achieves successful reduction in the majority of cases while avoiding the need for surgical intervention [16].

Nevertheless, complications may arise during treatment. These include femoral nerve palsy, avascular necrosis of the femoral head, inferior or posterior dislocation of the hip, and failure of reduction [17-19]. Among these, obturator (inferior) dislocation is considered a rare complication.

Obturator dislocation during Pavlik harness treatment has been described in the literature but remains uncommon [10-12]. Several authors have suggested that excessive hip flexion combined with inadequate abduction may predispose the femoral head to inferior displacement through the obturator region. Improper harness positioning or lack of early follow-up may contribute to this complication. Also, the morphological features of the patient, such as excessive acetabular dysplasia and/or joint hyperlaxity, may contribute to this complication.

In the present case, the harness was applied with increased flexion to maintain reduction. This positioning may have contributed to the inferior displacement of the femoral head. Early follow-up imaging using the anterior ultrasound approach enabled prompt identification of the abnormal hip position [13].

Early detection is essential because delayed recognition may lead to further complications, including persistent dysplasia, avascular necrosis, and long-term joint deformity. Once an obturator dislocation is identified, the Pavlik harness should be discontinued immediately, and alternative treatment strategies should be considered.

In our patient, closed reduction followed by spica casting successfully restored the position of the femoral head. However, despite appropriate treatment, the patient subsequently developed persistent dysplasia, femoral head fragmentation, and features consistent with coxa plana. These findings illustrate the potential for a cascade of complications following early treatment failure or atypical displacement patterns.

Long-term monitoring is therefore essential in patients with complicated DDH, as residual dysplasia may require surgical correction later in childhood [20]. In this case, a Salter innominate osteotomy is planned to address the remaining acetabular deficiency.

## Conclusions

Obturator dislocation of the hip is a rare but significant complication of Pavlik harness treatment for DDH. Early recognition through careful clinical and imaging follow-up is essential to prevent further complications.

This case emphasizes the importance of proper harness positioning, close monitoring during treatment, and prompt intervention when atypical displacement patterns are detected. Even with timely management, patients may experience a prolonged and complex treatment course requiring long-term follow-up and potential surgical correction.
